# Correction to “Enhanced Clearance of HIV‐1 Broadly Neutralizing Antibody VRC07‐523‐LS During Viremia: Influences on Trial Design and Analysis”

**DOI:** 10.1111/cts.70530

**Published:** 2026-03-18

N. M. Smith, B. M. Ho, K. J. Bar, et al., “Enhanced Clearance of HIV‐1 Broadly Neutralizing Antibody VRC07‐523‐LS During Viremia: Influences on Trial Design and Analysis,” *Clinical and Translational Science* 19, no. 2 (2026): e70488, https://doi.org/10.1111/cts.70488.

In the article cited above, Michael Pensiero was incorrectly included in the author list. The correct author list is shown below.

Nicholas M. Smith, Brian M. Ho, Katharine J. Bar, Lucio Gama, Gabrielle Dziubla, Richard A. Koup, Leonid Serebryannyy, Marina Caskey, Timothy J. Wilkin, Troy D. Wood, Gene D. Morse, Charles S. Venuto, Ray Cha, Qing Ma

We regret this error.

